# Relative and Quantitative Rhizosphere Microbiome Profiling Results in Distinct Abundance Patterns

**DOI:** 10.3389/fmicb.2021.798023

**Published:** 2022-01-24

**Authors:** Hamed Azarbad, Julien Tremblay, Luke D. Bainard, Etienne Yergeau

**Affiliations:** ^1^Evolutionary Ecology of Plants, Faculty of Biology, Philipps-University Marburg, Marburg, Germany; ^2^Energy, Mining and Environment, National Research Council Canada, Montréal, QC, Canada; ^3^Swift Current Research and Development Centre, Agriculture and Agri-Food Canada, Swift Current, SK, Canada; ^4^Centre Armand-Frappier Santé Biotechnologie, Institut National de la Recherche Scientifique, Laval, QC, Canada

**Keywords:** qPCR, quantitative, microbiome, amplicon sequencing, stress history

## Abstract

Next-generation sequencing is one of the most popular and cost-effective ways of characterizing microbiome in multiple samples. However, most of the currently available amplicon sequencing approaches are limited, as they result in relative abundance profiles of microbial taxa, which does not represent actual abundance in the environment. Here, we combined amplicon sequencing (16S rRNA gene for bacteria and ITS region for fungi) with real-time quantitative PCR (qPCR) to characterize the rhizosphere microbiome of wheat. We show that changes in the relative abundance of major microbial phyla do not necessarily follow the same pattern as the estimated quantitative abundance. Most of the bacterial phyla linked with the rhizosphere of plants grown in soil with no history of water stress showed enrichment patterns in their estimated absolute abundance, which was in contradiction with the trends observed in the relative abundance data. However, in the case of the fungal groups (except for *Basidiomycota*), such an enrichment pattern was not observed and the abundance of fungi remained relatively unchanged under different soil water stress history when estimated absolute abundance was considered. Comparing relative and estimated absolute abundances of dominant bacterial and fungal phyla, as well as their correlation with the functional processes in the rhizosphere, our results suggest that the estimated absolute abundance approach gives a different and more realistic perspective than the relative abundance approach. Such a quantification approach provides complementary information that helps to better understand the rhizosphere microbiomes and their associated ecological functional processes.

## Introduction

It is becoming increasingly evident that microorganisms, whether transmitted maternally (from parent to offspring) or environmentally (through the uptake of microbes from the environment), can strongly influence the biology of their host plants (Quiza et al., 2015; Agoussar and Yergeau, 2021). Over the past decades, due to the rapid advances in DNA sequencing technologies, our knowledge of the diversity and evolution of plant-associated microbiome has been greatly improved (Busby et al., 2017; Compant et al., 2019). However, the accurate characterization of microorganisms has been a challenging problem. Amplicon sequencing of marker genes such as the 16S rRNA gene and internal transcribed spacer (ITS) is appreciated as one of the most popular and cost-effective ways of surveying microbiomes in many samples of various types. Despite the clear advantages and potential for using marker gene sequencing, it has important biases and limitations, which should be considered more carefully when characterizing microbial communities based on amplicon data.

Most of the currently available amplicon sequencing approaches are inherently limited and produce compositional data, often presented as the relative abundance of microbial taxa (fraction of total reads), and do not consider inter-sample variations in microbial loads (Props et al., 2017). Thus, compositional data are constrained, and the biological interpretation of such datasets can be misleading (Vandeputte et al., 2017). This is particularly important when substantial differences in total microbial biomass between samples are expected, such as in climate change experiments where stressed samples (e.g., drought, heat, and salinity stresses) are compared to control samples. We know from previous studies that both bacterial and fungal species may show different response patterns in water-limited environments such as sensitive, tolerant and opportunistic (Evans and Wallenstein, 2012; Meisner et al., 2018), that ultimately shape community structure and many ecosystem functions. If, for instance, a single opportunistic bacterial species increase in absolute abundance (counts) under water stress, this will result (i) in an increase in its relative abundance (ratios) within the community and (ii) in a decrease in the abundance of all other species due to compositionality effects (Morton et al., 2017; Vandeputte et al., 2017), which is independent of ecological processes governing community profiles (Stämmler et al., 2016).

In their recent study, Alteio et al. (2021) discussed the possible technical challenges and limitations of amplicon sequencing and how compositionality may influence the integration of relative abundance data in soil microbiome research (Alteio et al., 2021). Different approaches have been proposed to link with amplicon sequencing to quantitively evaluate microbiomes such as qPCR (Zhang et al., 2017; Jian et al., 2020), flow cytometry that would allow counting microbial cells (Vandeputte et al., 2017), and the application of an internal standard (Tourlousse et al., 2016; Palmer et al., 2018; Tkacz et al., 2018). The advantage of incorporating qPCR with amplicon sequencing data has been successfully assessed using fecal samples (Jian et al., 2020), grassland soil from the Tibet Plateau (Zhang et al., 2017), and soil from a contaminated area (steel mill) in Fujian Province, China (Lou et al., 2018). However, empirical evidence is still limited, particularly in soil microbiomes from agricultural lands with contrasting soil water stress histories where substantial differences in microbial biomass are expected. In addition, in the context of absolute quantification of microbiome abundances, previous studies have mainly focused on the bacteria, without considering fungal communities.

The purpose of this study was to test the usefulness of relative and estimated absolute abundances of bacterial and fungal communities associated with the rhizosphere of wheat under historical and contemporary soil water stress. To address this, we grew four wheat genotypes (two with recognized drought resistance and two without) in soils with more than 40 years of exposure to different irrigation management histories (irrigated and non-irrigated) and exposed to various contemporary soil water limitations. Previous work from our team showed that soil stress history had a strong effect on the abundance of bacteria and fungi (assessed using qPCR) in the rhizosphere under the contemporary water stress (Azarbad et al., 2018). In another study, based on the same set-up, we showed that historical soil microbial water stress restructured bacterial and fungal communities in the rhizosphere of wheat plants (Azarbad et al., 2020). Here, we took advantage of prior information to integrate amplicon sequencing and qPCR data in order to consider inter samples differences in microbial biomass and quantitatively characterize the rhizosphere microbiomes. More specifically, we wanted to determine whether relative and quantitative abundances approaches would result in a similar pattern and which approach is more closely linked to the functional processes in the rhizosphere. One of the first priorities in managing the agroecosystem is to optimize important ecosystem functions such as carbon and nitrogen cycling (Oliver et al., 2015; Piton et al., 2021). Therefore, in this study soil respiration rate (CO_2_ production) was assessed as a measure of the rhizosphere functional response.

## Materials and Methods

### Soil Sample Collection and Experimental Design

Twenty soil samples were collected in April 2016 from the top layer (0–30 cm) of two experimental agricultural wheat fields located at the Swift Current Research and Development Centre (Agriculture and Agri-Food Canada) in Swift Current, SK, Canada. Although adjacent, these fields were managed differently since 1981 in such a way that one was irrigated (IR) during the wheat growing season and the other was not (NI), resulting in contrasting soil water stress histories. Soil samples were transported to the laboratory, mixed, homogenized (to obtain a representative soil for each wheat field), and sieved (2 mm). Three sub-samples of each soil stress history were kept at –20°C as “T0” for DNA extraction and downstream analysis. Soil samples were placed in a plant grow room to acclimatize the microbes to the new environment. After one month of incubation, on Jun 19th, 2016, eight seeds of two wheat genotypes with recognized resistance to water stress (*Triticum aestivum* cv. AC Barrie and *Triticum turgidum* subsp. *Durum* cv. Strongfield) and two without (*Triticum aestivum* cv. AC Nass and *Triticum aestivum* cv. AC Walton) were sown in pots (14.5 cm high × 19 cm diameter) containing 700 g of each type of soil (dry weight equivalent). Pots were placed in a growth room in a complete randomized block design. The 16 h light and 8 h dark photoperiods with an 800 μmol m^–2^ s^–1^ photon flux density and a steady temperature of 23 ± 1°C were applied throughout the experiment.

**FIGURE 1:**
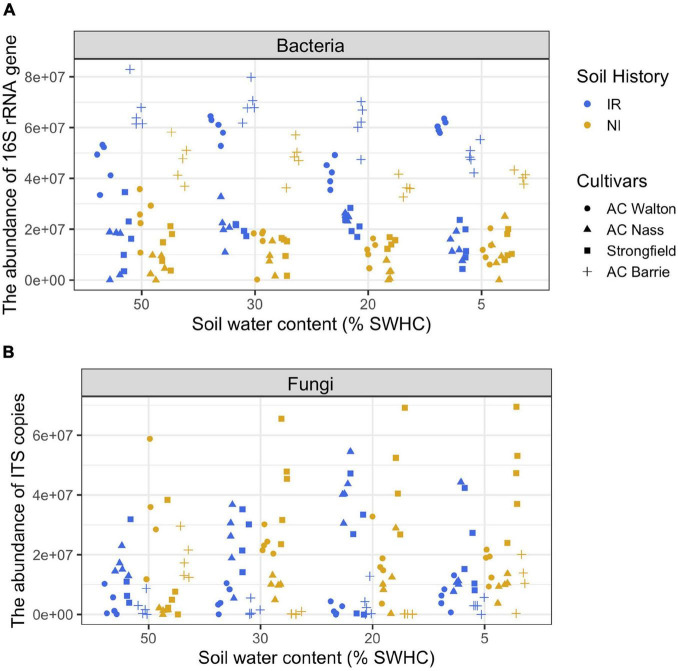
The abundance of **(A)** bacterial 16S rRNA gene (copies g^– 1^ soil dry weight) and **(B)** fungal ITS region (copies g^– 1^ soil dry weight) in the rhizosphere of four wheat genotypes grown in soil with two contrasting stress histories (Soil history) under different contemporary water stress (SWHC).

During the first 4 weeks of the growth, plants were kept under well-watered conditions (50% soil water holding capacity, SWHC), then they were subjected to 5–8% SWHC, 20% SWHC, and 30% SWHC, while controls were kept at 50% SWHC. To keep the target soil water content (SWC), the pot weights were measured every day. If needed, water was added by taking into account different biomass accumulation for each wheat genotype using pots without plants as a control. Rhizosphere samples were collected (4 wheat genotypes × 2 soil history types × 4 SWHC × 5 replicates = 160 samples) at the end of the experiment (after 4 weeks of exposure to the different SWHC). The following parameters were measured in all collected rhizosphere samples: CO_2_ production, microbial community structure, and the total abundance of bacteria and fungi. The experimental design, CO_2_ production measurements, real-time quantitative PCR assays (qPCR), and amplicon sequencing assays have been previously published (Azarbad et al., 2018, 2020). Some of these methods are described below. Detailed information regarding amplicon library construction and sequencing is provided as Supplementary Material. For the purpose of discussion, qPCR data are presented in Figure 1.

### CO_2_ Production Measurements

Detailed information on how CO_2_ production was determined has been previously published (Azarbad et al., 2018). Briefly, microcosms including rhizosphere samples (5 g dry weight soil), were tightly closed with rubber septum caps. To assess CO_2_ production, headspace samples (10 cm^3^) were collected with a syringe and injected into a gas chromatograph injection port (Agilent 7890 A, Agilent Technologies, Santa Clara, CA, United States). CO_2_ production was measured every 24 h for 3 days at 24°C. Using linear regression, rhizosphere CO_2_ production was calculated from the slope of the change in CO_2_ concentration following several measurement points (10, 1,440, and 2,880 min). Empty microcosms (without rhizosphere samples) were used as the black samples, and no CO_2_ production was detected.

### DNA Extraction and qPCR Assays

Genomic DNA was extracted from 0.5 mg of rhizosphere soil using a phenol-chloroform extraction method (Dellaporta et al., 1983). Further details on DNA extraction can be found in Azarbad et al. (2018). The total abundance of bacterial (16S rRNA genes) and fungal (ITS1 region) communities associated with the rhizosphere were quantified using SyBrGreen real-time quantitative PCR assays (qPCR) with the primers listed below (same as those used for amplicon sequencing). Briefly, the qPCR reactions were carried out using a RotorGene 6000 machine (Corbett Research, Mortlake, NSW, Australia) with SsoAdvanced™ Universal SYBR Green kits (Biorad, Hercules, CA, United States). We have performed several test runs including a wide range of dilution of extracted DNA to (1) find out the range of linear amplification of extracted DNA to ensure that all samples are in the expected scale based on the standard curve and (2) to reduce qPCR inhibition. The standard curve ranging from 0 to 10 exp 7, copies of the standard plasmid DNA were prepared using *Escherichia coli* 25922 for bacteria (Bruce et al., 1992) and *Pichia scolyti* for fungi (Martin and Rygiewicz, 2005). We have chosen 10 random samples and then prepared 10, 50, 100, 200, and 400-fold diluted and non-diluted DNA extracts. We also included two blank samples (nuclease-free water) as controls. Based on these results, the optimum dilutions were selected if the amplification products were between expected ranges (above the minimum detection limit to the middle point of the linear range of standards). As a result, DNA fragments corresponding to the rhizosphere were diluted 10 times. Each qPCR mix consisted of 4.2 μl sterilized water, 10 μl SYBR green master mix, 0.4 μl of each primer (0.4 pmoles/μl) and 5 μl of diluted template DNA for a final reaction volume of 20 μl. Similar to amplicon sequencing analysis, for the qPCR assays bacterial 16S rRNA gene universal primers 520F (5′-AGCAGCCGCGGTAAT-3′) and 799R (5′-CAGGGTATCTAATCCTGTT-3′) (Edwards et al., 2008), and the fungal ITS1F (5′-CTTGGTCATTTAGAGGAAGTAA-3′) and 58A2R (5′-CTGCGTTCTTCATCGAT-3′) (Martin and Rygiewicz, 2005) were used. The PCR conditions consisted of an initial denaturation step at 95°C for 5 min followed by 30 cycles of denaturation at 95°C for 30 s, annealing at 57°C for 30 s and elongation at 72°C for 30 s. Fluorescence was measured at the end of each cycle at the elongation step. A melt curve analysis was done to verify the specificity of the amplicons. The qPCR cycle threshold (Ct) values are presented in Supplementary Figure 1.

### Statistical Analyses

Statistical analyses were carried out using R (The R Foundation for Statistical Computing) and the PAST program (Hammer et al., 2001). In this study, absolute gene copy numbers based on qPCR assays varied between the “T0” samples (that is three subsamples of the sieved soil before wheat seeding) for both bacteria (IR soils: 3.9 × 10^7^ copies and NI soils: 2.2 × 10^7^ copies g^–1^ soil-dry weight) and fungi (IR soils: 9.8 × 10^6^ copies and NI soils: 1.6 × 10^7^ copies g^–1^ soil-dry weight). To consider the inter-sample differences in the initial microbiome biomass, we measured the number of copies of the 16S rRNA gene and of the ITS region in each rhizosphere sample to estimate the absolute abundances of bacteria and fungi and to normalize amplicon sequencing data. Two datasets were produced: OTU relative abundance (fraction of total reads) and absolute OTU abundance, which was estimated by multiplying the OTU relative abundance matrix by the corresponding abundance of 16S rRNA gene and ITS region obtained by qPCR quantifications, as previously suggested (Zhang et al., 2017; Lou et al., 2018; Jian et al., 2020). Since soil history was previously identified as the main factor structuring rhizosphere microbial communities (Azarbad et al., 2018, 2020; Figure 1 and Supplementary Figure 2), we focused on this factor for the needs of our demonstration. Still, similar conclusions could be reached by focusing on cultivar or SWC effects. Principal coordinate analyses (PCoA) based on Bray–Curtis dissimilarity were performed to visualize the effect of soil history, wheat genotype, and SWC on rhizosphere-associated microbial community composition. The effects of the experimental factors and their possible interactions on microbial community composition were assessed using Permanova (with 1,000 permutations). To investigate the possible impact of soil history on the relative and estimated absolute abundance of the most abundant bacterial and fungal phyla associated with the rhizosphere of wheat genotypes, an analysis of variance (ANOVA) was performed. Because of the primary role of bacteria in soil organic matter decomposition, Pearson correlation tests were performed between the estimated absolute and relative abundances of the dominant bacterial phyla and CO_2_ production to verify which approach is most closely related to the functional processes in the rhizosphere.

## Results and Discussion

### Relative and the Estimated Absolute Abundance Data Give a Contrasting Pattern

PCoAs based on relative abundance data revealed that soil history was the primary factor shaping bacterial (Supplementary Figure 2A) and fungal (Supplementary Figure 2C) communities associated with the rhizosphere of wheat genotypes. Permanova analyses confirmed PCoAs patterns and showed that soil history was the main source of variation (higher F-ratio, Supplementary Table 1). However, when PCoA based on estimated absolute data was performed, we observed notably different patterns such that, besides the strong effect of soil history, the effect of SWC and wheat genotype became more evident (Supplementary Figures 2B,D). Permanova analyses corroborated this finding, indicating a higher F-ratio for the genotype and SWC effect for the estimated absolute dataset (Supplementary Table 1). In the following parts, to better differentiate relative and the estimated absolute abundance pattern, particular attention is paid to the soil history.

**FIGURE 2:**
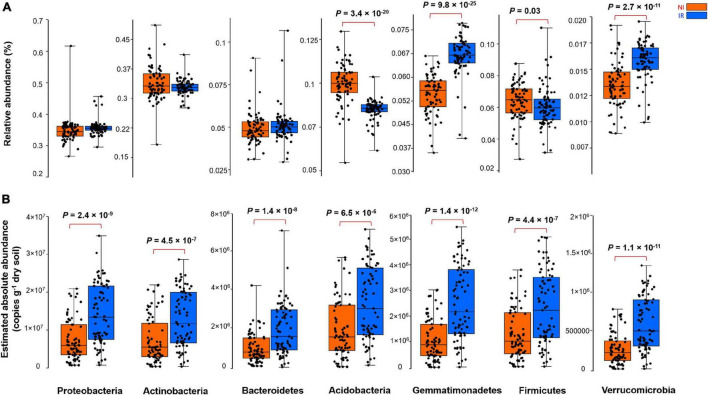
The effect of soil history on **(A)** the relative and **(B)** estimated absolute abundance of different bacterial phyla associated with the rhizosphere of four wheat genotypes grown in Saskatchewan soils with a water stress history (NI) or with no history of water stress (IR) exposed to four levels of soil water content. Dots are values for individual observations, the horizontal lines in boxes are representative of the median, the upper and lower part of boxes indicating 75th and 25th quartiles, and whiskers on the boxes showing 1.5 × the interquartile range. ANOVA tests comparing the abundance of each phylum between IR and NI soils.

**FIGURE 3:**
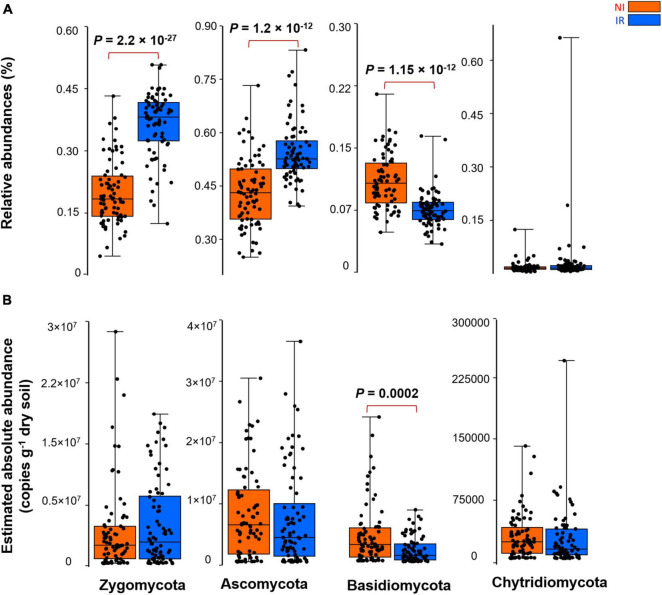
The effect of soil history on **(A)** the relative and **(B)** estimated absolute abundance of different fungal phyla associated with the rhizosphere of four wheat genotypes grown in Saskatchewan soils with a water stress history (NI) or with no history of water stress (IR) exposed to four levels of soil water content. Dots are values for individual observations, the horizontal lines in boxes are representative of the median, the upper and lower part of boxes indicating 75th and 25th quartiles, and whiskers on the boxes showing 1.5 × the interquartile range. ANOVA tests comparing the abundance of each phylum between IR and NI soils.

By comparing the relative and the estimated absolute abundances of dominant bacterial and fungal phyla associated with the rhizosphere, we observed completely different and sometimes contradictory trends. For instance, the relative abundances of *Acidobacteria* and *Firmicutes* were significantly higher in the rhizosphere of plants growing in the non-irrigated soil as compared to the irrigated soil (Figure 2A). In contrast, the estimated abundances of *Proteobacteria*, *Actinobacteria*, *Bacteroidetes*, *Acidobacteria*, *Gemmatimonadetes*, *Firmicutes*, and *Verrucomicrobia* were significantly higher in the rhizosphere of plants grown in irrigated soil when compared to non-irrigated soils (Figure 2B). On the other hand, *Gemmatimonadetes* and *Verrucomicrobia* had significantly higher relative abundances in the rhizosphere of plants grown in irrigated soil than non-irrigated soils, coherent with the picture observed for estimated abundances (Figure 2A). It has been shown previously that the relative abundance of *Actinobacteria* increases under dry conditions and this phylum becomes a dominant group of bacteria in the soil environment (Naylor et al., 2017; Meisner et al., 2018; Preece et al., 2019). However, based on our study, when the estimated absolute abundance was considered, we observed the opposite pattern where the rhizosphere of plants growing in the soil with no history of water stress harbored significantly more *Actinobacteria* as compared with the rhizosphere of plants growing in the soil with a water stress history (Figure 2B). There were also inconsistent trends between the relative and estimated absolute abundances for fungal phyla (Figure 3). For instance, the relative abundance of *Zygomycota* and *Ascomycota* increased in the rhizosphere of plants grown in irrigated soils, but this pattern was absent when looking at the estimated abundances (Figures 3A,B). In contrast, both relative and estimated abundances agreed that a history of water stress significantly increases the abundance of *Basidiomycota* in the rhizosphere of wheat (Figures 3A,B).

In the present study, absolute gene copy numbers (qPCR data) were significantly different between the two soils before starting the experiment, which is one of the many cases for which analyses of microbial communities based on relative abundance is unlikely to reflect actual community patterns (Stämmler et al., 2016; Props et al., 2017; Vandeputte et al., 2017; Zhang et al., 2017; Lundberg et al., 2020) and associated ecosystem processes. Here, when estimated absolute abundances were considered, we observed notable differences from the relative abundance data, and new, often contradictory, trends became visible. It is relatively easy to understand why these differences occur: for example, if a single drought-tolerant bacterial species increases its absolute abundance under water stress, this will increase its relative abundance and a concomitant decrease in the relative abundance of all other species, even though their absolute abundance did not change. Such compositional effects may not fully reflect actual microbial profiles and their associated ecological functional processes.

### The Estimated Absolute Abundance Gives a More Realistic View of the Impact of Soil Water Stress History on the Rhizosphere Microbiome Profiles

As shown in the previous section, depending on relative and estimated absolute abundance approaches, an inconsistent pattern was evident in the response of rhizosphere microbiome associated with plants grown in soil with contrasting soil water stress histories. For instance, most of the bacterial phyla linked with the rhizosphere of plants grown in soil with no history of water stress showed enrichment patterns in their estimated absolute abundance (Figure 2). However, in the case of the fungal group (except for *Basidiomycota*), such an enrichment pattern was not observed and the abundance of fungi showed no significant change under different soil water stress histories when estimated absolute abundance was considered (Figure 3). It is well known from previous research that fungi have strong cell walls (Schimel et al., 2007) and a hyphal network (Khalvati et al., 2005) which help them to better withstand drought conditions than bacterial (Manzoni et al., 2012; Fuchslueger et al., 2014). These results indicate that the estimated absolute abundance based on the qPCR method gives a more realistic picture of changes in microbial profile under water-limited environments.

### Relative and Quantitative Abundances and CO_2_ Production

To determine which approach was more closely linked to functional processes in the rhizosphere, we correlated relative and absolute abundances of dominant bacterial phyla with CO_2_ production, which was previously measured (Azarbad et al., 2018). When the estimated absolute abundance was used, Pearson correlations showed no significant correlations between bacterial phyla and CO_2_ production. However, when relative abundance data were used, we observed significant negative (*Actinobacteria*: *r* = −0.361, *p* = < 0.001) and positive (*Acidobacteria*: *r* = 0.158, *p* = 0.048; *Gemmatimonadetes*: *r* = 0.272, *p* = 0.001; *Proteobacteria*: *r* = 0.277, *p* = < 0.001) correlations between bacterial phyla and CO_2_ emissions (Table 1).

**TABLE 1:** Correlation tests between relative and absolute abundances of the most abundant bacterial phyla associated with the rhizosphere of four wheat genotypes grown in Saskatchewan soils with a water stress history (NI) or with no history of water stress (IR) exposed to four levels of soil water content vs. CO_2_ production.

	CO_2_ production			

	Relative abundance		Quantitative abundance	
	*R*	*P*-value	*R*	*P*-value
Acidobacteria	0.158	**0.048**	0.101	0.208
Actinobacteria	–0.361	** < 0.001**	0.040	0.615
Bacteroidetes	–0.036	0.656	0.061	0.451
Firmicutes	–0.079	0.327	0.051	0.528
Gemmatimonadetes	0.272	**0.001**	0.104	0.193
Proteobacteria	0.277	** < 0.001**	0.093	0.249
Verrucomicrobia	0.071	0.378	0.078	0.331

*Bold P-values are significant at P < 0.05.*

Using real-time PCR, we previously showed that the microbial abundance is quite stable across SWHC treatments for both soil history types (Azarbad et al., 2018). However, CO_2_ emissions were severely reduced under low water content, which is clearly due to a change in the activity of the microbial community, rather than a massive death and reduction of the abundance of the microbial communities. The estimated absolute data supported this observation as no correlation between the abundance of the dominant bacterial phyla and CO_2_ production was detected. Conversely, in the case of the relative abundance approach, significant correlations between many taxa and CO_2_ production were found. These correlations were most probably spurious and due to shifts in the microbial community composition with decreasing water content. Some results were even non-sensical, as negative correlations were found, which would be interpreted as if soil respiration would decrease when some taxa become more abundant.

## Conclusion and Future Directions

The objective of this study was to differentiate the change in the relative and estimated absolute abundances of the rhizosphere of wheat plants when grown in soil with contrasting soil water stress histories under contemporary water limitations. With a commonly used qPCR protocol for both bacteria and fungi, this study demonstrated the usefulness of incorporating changes in microbial biomass to rhizosphere microbiome evaluation. Comparing relative and estimated absolute abundances of dominant bacterial and fungal phyla, as well as their correlation with CO_2_ production in the rhizosphere, allowed us to conclude that the estimated absolute quantification provides a more realistic view of the impact of soil water stress history on the rhizosphere microbiome profiles. This conclusion is consistent with a study by Lou et al. (2018), who reported that qPCR is an accurate approach to quantitively evaluate the absolute abundance of genes, thus integrating the qPCR with high-throughput sequencing helped to better characterize the actual change in microbial abundance. Since both qPCR and sequencing approaches were performed on the same DNA extract with the same set of primers, they shared the same methodological limitations (e.g., amplification efficiency and the specificity of primers) thereby making the qPCR and sequencing data compatible (Dannemiller et al., 2014; Props et al., 2017; Jian et al., 2020). In line with this, Jian et al. (2020) discussed the advantage and potential biases of microbiome data with regards to linking qPCR with amplicon-based sequencing data. In their study, they pointed out that since the qPCR does not add additional biases, which already exist in the amplicon sequencing approach, it can be considered as an advantage.

It is important to mention that the primers used in this study are designed to exclude plant mitochondrial and chloroplast DNA, which was one of the main criteria to select these sets of primers. We acknowledge that contamination might still occur due to the homology between bacterial 16S rRNA genes and plant material. However, since the focus of this study was only on the soil and rhizosphere microbiomes, this should have a minor impact on the resulting data. We believe that this approach, with its limitations, is better than reporting only relative abundance data (Dannemiller et al., 2014; Jian et al., 2020).

In this study, we performed qPCR of the V3-V4 hypervariable region of the 16S rRNA gene (which serves as a standard marker to assess total bacterial biomass) and of the ITS1 region to assess fungal biomass. To provide better insights into the abundance of specific taxa, another approach would be to perform qPCR by using specific primers for various bacterial and fungal taxa. Therefore, we highly encourage further studies to perform qPCR with taxa-specific primers which would provide the ability to track whether a given taxa increased its absolute abundance while other taxa remained the same without the need for sequencing. Other promising approaches such as flow cytometry (Props et al., 2017), adding a known number of 16S rRNA gene copies of exogenous bacteria into the samples before DNA extraction to normalize endogenous bacterial counts (Stämmler et al., 2016) or synthetic spike-in standards (Tourlousse et al., 2016; Palmer et al., 2018; Tkacz et al., 2018) could also be applied to estimate microbial loads.

In summary, this study allowed us to quantitatively evaluate the differences between the rhizosphere microbiome of wheat plants growing in soil with contrasting long-term water management history. We showed that quantitative microbiome profiling provides a contrasting picture of the response of rhizosphere microbial communities to soil water stress legacy, which appeared to be better aligned to actual ecosystem processes. Such a quantification approach provides complementary information that helps to better interpret changes in the abundance of microbial taxa, which is critical when substantial differences in total microbial biomass between samples are expected.

## Data Availability Statement

Raw sequencing data sets are available in the NCBI Sequence Read Archive (SRA) under the BioProject accession PRJNA526458.

## Author Contributions

HA and EY designed the study. HA planned and performed the experiment. LB contributed in soil collections. JT performed the bioinformatic analyses and bioinformatic methods writing. HA analyzed the data and wrote the manuscript with the help of EY. All authors participated in reviewing and editing the final text.

## Conflict of Interest

The authors declare that the research was conducted in the absence of any commercial or financial relationships that could be construed as a potential conflict of interest.

## Publisher’s Note

All claims expressed in this article are solely those of the authors and do not necessarily represent those of their affiliated organizations, or those of the publisher, the editors and the reviewers. Any product that may be evaluated in this article, or claim that may be made by its manufacturer, is not guaranteed or endorsed by the publisher.
