# Supplementary material for: Relative and Quantitative Rhizosphere Microbiome Profiling Results in Distinct Abundance Patterns
Source: Front Microbiol. 2022 Jan 24;12:798023. doi: 10.3389/fmicb.2021.798023 (PMC8819139; doi:10.3389/fmicb.2021.798023)
Supplement: Supplementary file 1 [file Data_Sheet_1.docx]

**Supplementary Methods**

*Amplicon library construction and sequencing*

Sequencing libraries were created as described previously in Yergeau *et al*. (2015) based on a dual-indexed strategy following the “16S Metagenomic Sequencing Library preparation” Illumina guide (Part #15044223 Rev. B). Similar to qPCR analysis, for bacterial 16S rRNA gene the V3-V4 hypervariable region was amplified using the universal primers 520F (5’-AGCAGCCGCGGTAAT-3’) and 799R (5’-CAGGGTATCTAATCCTGTT-3’) (Edwards *et al*., 2008), and for fungi the ITS1 region was amplified using ITS1F (5’-CTTGGTCATTTAGAGGAAGTAA-3’) and 58A2R (5’-CTGCGTTCTTCATCGAT-3’) (Martin and Rygiewicz, 2005). Samples were pooled separately for fungi and bacteria and submitted for 2 × 250 bp Illumina MiSeq sequencing at the McGill University and Genome Québec Innovation Centre (Montréal, Canada). Sequence data were analysed following procedures described in Tremblay *et al*. (2015). Briefly, raw reads were controlled for quality. The remaining high-quality reads and free of sequencing adapters artifacts were dereplicated at 100% identity and clustered/denoised at 99% (DNAclust v3) (PMID:21718538). Clusters of less than three reads were discarded, and the remaining clusters were scanned for chimeras using UCHIME, first in de novo mode then in reference mode (Edgar *et al*., 2011). The remaining clusters were clustered at 97% identity (DNAclust v3) to produce OTUs. For 16S data types, taxonomy assignment of resulting OTUs was performed using the RDP classifier (Wang *et al*., 2007) with a modified Greengenes training set built from a concatenation of the Greengenes database v13_5 (DeSantis *et al*., 2006), and Silva eukaryotes 18S r128 (Quast *et al*., 2013). For ITS data, the taxonomic assignment was done with the RDP classifier using a training set generated from the Unite database (sh_refs_qiime_ver7_dynamic_20.11.2016) (Kõljalg *et al*., 2013). Raw data sets are available in the NCBI Sequence Read Archive (SRA) under the BioProject accession PRJNA526458.

**References**

Yergeau E, Bell TH, Champagne J, Maynard C, Tardif S, Tremblay J, Greer CW. Transplanting Soil Microbiomes Leads to Lasting Effects on Willow Growth, but not on the Rhizosphere Microbiome. *Front Microbiol* 2015;**6**:1436.

Tremblay J, Singh K, Fern A, Kirton ES, He S, Woyke T, Lee J, Chen F, Dangl JL, Tringe SG. Primer and platform effects on 16S rRNA tag sequencing. *Front Microbiol* 2015;**6**:771.

Wang Q, Garrity GM, Tiedje JM, Cole JR. Naive Bayesian classifier for rapid assignment of rRNA sequences into the new bacterial taxonomy. *Appl Environ Microbiol* 2007;**73**:5261–5267.

DeSantis TZ, Hugenholtz P, Larsen N, Rojas M, Brodie EL, Keller K, et al. Greengenes, a chimera-checked 16S rRNA gene database and workbench compatible with ARB. *Appl Environ Microbiol* 2006;**72**:5069–5072.

Quast C, Pruesse E, Yilmaz P, Gerken J, Schweer T, Yarza P, et al. The SILVA ribosomal RNA gene database project: improved data processing and web-based tools. *Nucleic Acids Res* 2013; **41**: 590–596.

Kõljalg U, Nilsson RH, Abarenkov K, Tedersoo L, Taylor AFS, Bahram M, et al. Towards a unified paradigm for sequence-based identification of fungi. *Mol Ecol* 2013; **22**: 5271–5277.
