# Supplementary material for: Relative and Quantitative Rhizosphere Microbiome Profiling Results in Distinct Abundance Patterns
Source: Front Microbiol. 2022 Jan 24;12:798023. doi: 10.3389/fmicb.2021.798023 (PMC8819139; doi:10.3389/fmicb.2021.798023)
Supplement: Supplementary file 2 [file Data_Sheet_2.PDF]

Table S1. Permanova tests for the effects of soil history, soil water content and genotype, and their interactions on rhizosphere-associated microbial communities.

**Bacterial communities**				
	Relative		Quantitative	
**Factor**	F	P	F	P
**SWC**	0.15	**0.001**	0.21	**0.018**
**Genotype**	0.13	**0.001**	2.13	**0.001**
**Soil history**	3.52	**0.001**	3.69	**0.001**
**Block**	0.04	0.490	0.20	**0.011**
**SWC × Soil history**	0.07	**0.012**	0.16	0.082
**SWC × Genotype**	0.04	0.307	0.12	0.147
**Genotype × Soil history**	0.08	**0.014**	0.73	**0.001**
**SWC × Genotype × Soil history**	0.05	0.229	0.13	0.133
**Fungal communities**				
	Relative		Quantitative	
	F	P	F	P
**SWC**	0.25	**0.001**	0.39	**0.009**
**Genotype**	0.26	**0.001**	1.69	**0.001**
**Soil history**	9.84	**0.001**	4.66	**0.001**
**Block**	0.08	0.190	0.15	0.721
**SWC × Soil history**	0.13	**0.006**	0.26	0.103
**SWC × Genotype**	0.09	**0.042**	0.45	**0.001**
**Genotype × Soil history**	0.16	**0.007**	1.44	**0.001**
**SWC × Genotype × Soil history**	0.08	0.119	0.37	0.229

[a] Soil water content (SWC) refers to: high water content (50% soil water holding capacity, SWHC); moderate water content (30% and 20% SWHC) and low water content (5-8% SWHC).

[b] Genotype refers to AC Nass (*Triticum aestivum*), AC Walton (*Triticum aestivum*), AC Barrie (*Triticum aestivum*), Strongfield (*Triticum turgidum* subsp. *durum*)

[c] Soil history refers to soils with either an history of water stress (NI: non-irrigated soils from Saskatchewan) or with no recent history of water stress (IR: irrigated soils from Saskatchewan).

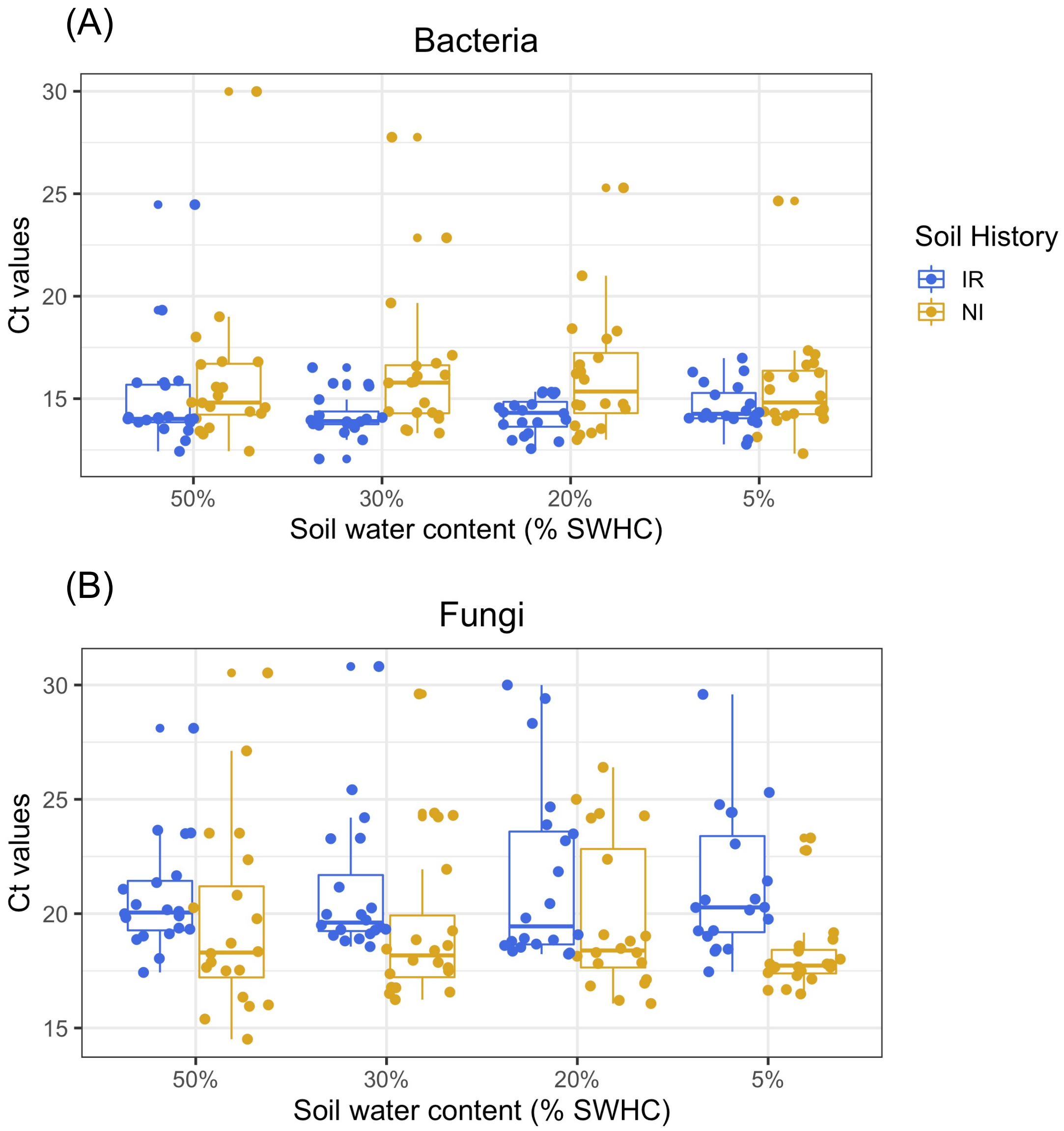

Fig S1. The Ct (cycle threshold) of qPCR data for (A) bacterial and (B) fungi in the rhizosphere of four wheat genotypes grown in soil with two contrasting stress histories (Soil history) under different contemporary water stress (SWHC).

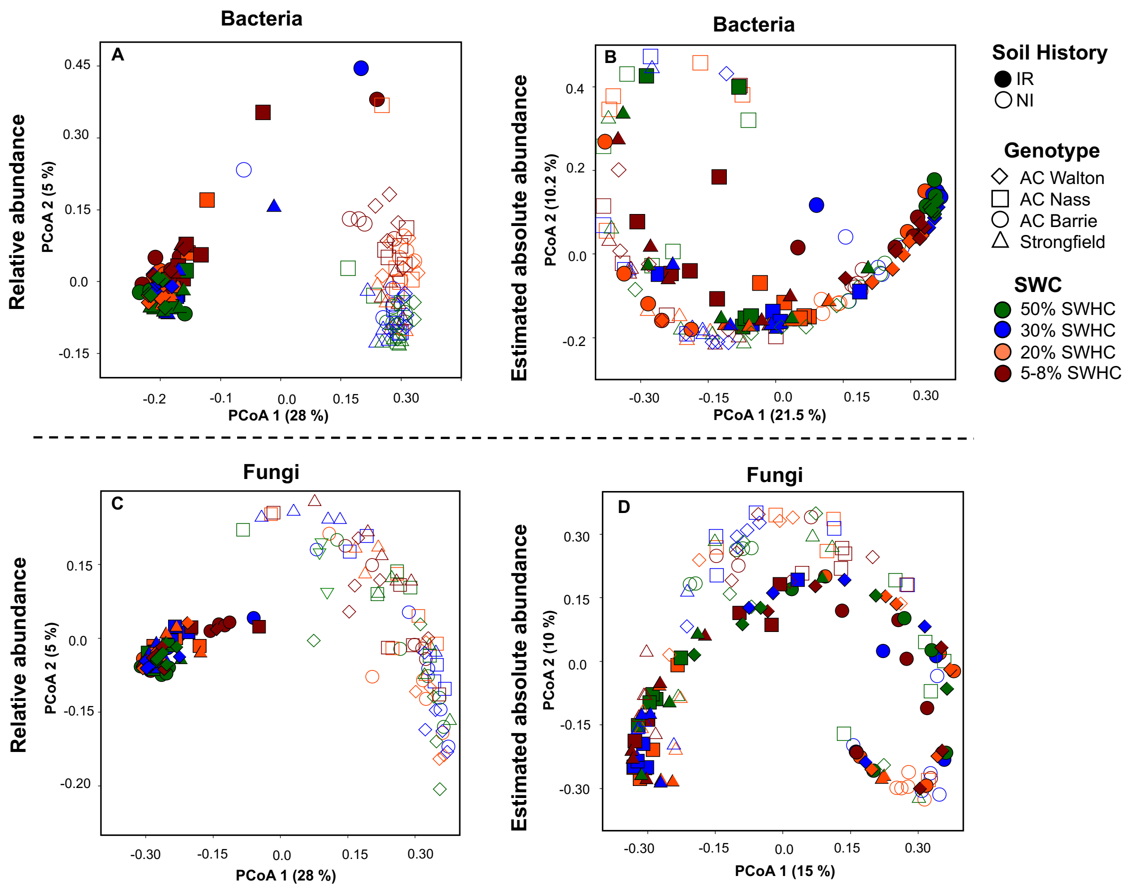

Fig. S2. Principal coordinate analyses (PCoA) of Bray–Curtis dissimilarity for relative abundance (A, C) and estimated absolute abundance (B, D) visualizing the impact of each of the experimental factors (Soil history, SWC, and wheat genotype) on bacterial and fungal communities associated with the rhizosphere.
